# The second complete chloroplast genome sequence of the *Viburnum erosum* (Adoxaceae) showed a low level of intra-species variations

**DOI:** 10.1080/23802359.2019.1698360

**Published:** 2019-12-13

**Authors:** Yun Gyeong Choi, Narae Yun, Jongsun Park, Hong Xi, Juhyeon Min, Yongsung Kim, Sang-Hun Oh

**Affiliations:** a Department of Biology, Daejeon University, Daejeon, The Republic of Korea;; b International Biological Material Research Center, Korea Research Institute of Bioscience and Biotechnology, Daejeon, The Republic of Korea;; c Infoboss Co., Ltd, Seoul, The Republic of Korea;; d InfoBoss Research Center, Seoul, The Republic of Korea

**Keywords:** *Viburnum*, chloroplast genome, *Viburnum erosum*, Adoxaceae, intraspecies variations

## Abstract

We presented the second complete chloroplast genome of the plant. The length of chloroplast genome is 158,587 bp, consisting of four subregions: 87,050 bp of LSC and 18,503 bp of SSC regions separated by a pair of 26,517 bp IR regions. It includes 129 genes (84 protein-coding genes, 8 rRNAs, and 37 tRNAs). A low-level of molecular variation within *Viburnum erosum* was found with 16 SNPs and 49 indels. The phylogenetic tree shows that the two accessions of *V. erosum* are clustered with *Viburnum japonicum* with no resolution between the species, suggesting that chloroplast genome in these species evolve slowly.

*Viburnum erosum* Thunb. is a common species in East Asia, characterized by having extrafloral nectaries on the abaxial surface of leaves and stipules in Adoxaceae (Choi and Oh 2019). As *V*. *erosum* is widely distributed from southern China, Taiwan, and Korea to Japan the species may be a model system to examine a geographical structure across the regions within the species. To evaluate the level of molecular variation at the genomic level within the species, we determined the second complete chloroplast genome sequence.

Plant sample of *V. erosum* was collected on Mt. Samsungsan (35°46′16.78′′N, 128°47′43.72′′E) in Gyeongsan-si, Gyeongsangbuk-do, Korea (voucher in the herbarium of Daejeon University (TUT); *Oh KBVE_01*). Molecular methods for DNA extraction, genome sequencing, and *de novo* assembly were done by the method described in Park, Choi, et al. (2019). Genome annotation was based on *V. erosum* chloroplast genome (MN218778; Park, Choi, et al. 2019) using Geneious R11 v11.0.5 (Biomatters Ltd, Auckland, New Zealand).

The chloroplast genome of *V. erosum* (GenBank accession number is MN641480) is 158,587 bp with four subregions: 87,050 bp of large single-copy (LSC), 18,503 bp of small single-copy (SSC) regions, and 26,517 bp of a pair of inverted repeats (IRs). It contains 129 genes (84 protein-coding genes, 8 rRNAs, and 37 tRNAs); 18 genes (7 protein-coding genes, 4 rRNAs, and 7 tRNAs) are duplicated in the IR regions. The overall GC-ratio of *V. erosum* was 38.1% and those in the LSC, SSC, and IR regions were 36.4, 32.0, and 43.0%, respectively.

Based on the alignment of two *V. erosum* genomes conducted by MAFFT 7.388, (Katoh and Standley 2013), 16 single nucleotide polymorphisms (SNPs) and 49 insertions and deletions (INDELs) were identified. There is one non-synonymous substitution in *ndhL* and *ycf1*, respectively, and one synonymous change in *psbB*. The remaining 13 SNPs are located in the non-coding regions. This low level of sequence variation is similar to intra-species variations of *Cucumis melo* (Zhu et al. 2016), *Chenopodium quinoa* (Maughan et al. 2019), *Coffea arabica* (Park, Kim, et al. 2019), *Artemisia fukudo* (Min et al. 2019), and *Dysphania pumilio* (Park and Kim 2019). Interestingly, numbers of SNPs and INDELs between *V. erosum* (Park, Choi, et al. 2019) and *V. japonicum* (Cho et al. 2018) are 18 and 36 (Figure 1(A)), which is lower than those between two *V. erosum* accessions and between *Salix koriyanagi* and *Salix gracilistyla* (Xi et al. 2019).

**Figure 1.**
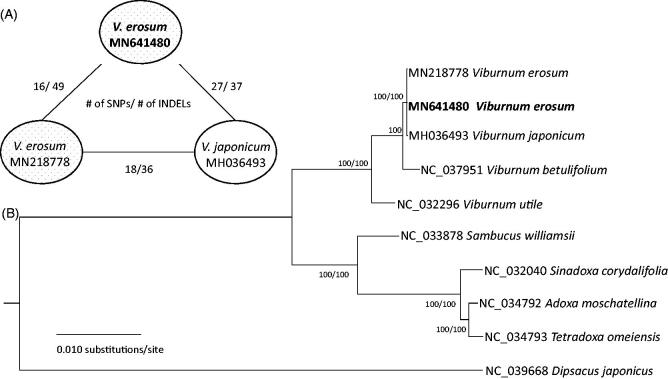
(A) Pairwise comparison of two *V. erosum* and *V. japonicum*. (B) A maximum-likelihood tree of based on Adoxaceae nine complete chloroplast genomes: *Viburnum erosum* (MN641480 in this study and MN218778), *Viburnum japonicum* (MH036493), *Viburnum betulifolium* (NC_037951), *Viburnum utile* (NC_032296), *Adoxa moschatellina* (NC_034792), *Sambucus williamsii* (NC_033878), *Sinadoxa corydalifolia* (NC_032040), and *Tetradoxa omeiensis* (NC_034793). The numbers above branches indicate bootstrap support values of maximum-likelihood and neighbor-joining phylogenetic trees. Numbers of single nucleotide polymorphisms (SNPs) and insertions and deletions (INDELs) between each pair are indicated along the branch.

Complete chloroplast genomes from nine chloroplast genomes of Adoxaceae and one outgroup species, *Dipsacus japonicus* (NC_039668), were included in the phylogenetic analysis using the neighbor-joining and maximum-likelihood methods. Sequence alignment and phylogenetic trees were constructed by using MEGA X (Kumar et al. 2018) with 10,000 and 1000 bootstrap repeats, respectively.

The phylogenetic tree shows that the two accessions of *V. erosum* are clustered with *V. japonicum* with no resolution between the species (Figure 1(B)). The phylogenetic tree shows that the chloroplast genomes of the two species are highly conserved, suggesting that the chloroplast genome in these species evolve slowly or the two species originated from their common ancestor very recently.
